# Headteachers’ prior beliefs on child health and their engagement in school based health interventions: a qualitative study

**DOI:** 10.1186/s13104-015-1091-2

**Published:** 2015-04-18

**Authors:** Charlotte Todd, Danielle Christian, Helen Davies, Jaynie Rance, Gareth Stratton, Frances Rapport, Sinead Brophy

**Affiliations:** College of Medicine, Swansea University, Swansea, SA2 8PP UK; College of Health and Human Science, Swansea University, Wales, SA2 8PP UK; Applied Sports Technology Exercise and Medicine Research Centre, College of Engineering, Swansea University, Wales, SA2 8PP UK

**Keywords:** Primary school, Qualitative, Headteacher, Children, Health, Beliefs

## Abstract

**Background:**

Schools play an important role in promoting the health of children. However, little consideration is often given to the influence that headteachers’ and school staff’s prior beliefs have on the implementation of public health interventions. This study examined primary school headteachers’ and school health co-ordinators’ views regarding child health in order to provide greater insights on the school’s perspective for those designing future school-based health interventions.

**Methods:**

A qualitative study was conducted using 19 semi-structured interviews with headteachers, deputy headteachers and school health co-ordinators in the primary school setting. All transcripts were analysed using thematic analysis.

**Results:**

Whilst many participants in this study believed good health was vital for learning, wide variance was evident regarding the perceived health of school pupils and the magnitude of responsibility schools should take in addressing child health behaviours. Although staff in this study acknowledged the importance of their role, many believed the responsibility placed upon schools for health promotion was becoming too much; suggesting health interventions need to better integrate school, parental and societal components. With mental health highlighted as an increasing priority in many schools, incorporating wellbeing outcomes into future school based health interventions is advocated to ensure a more holistic understanding of child health is gained.

**Conclusion:**

Understanding the health beliefs of school staff when designing interventions is crucial as there appears to be a greater likelihood of interventions being successfully adopted if staff perceive a health issue as important among their pupils. An increased dependability on schools for addressing health was expressed by headteachers in this study, highlighting a need for better understanding of parental, child and key stakeholder perspectives on responsibility for child health. Without this understanding, there is potential for certain child health issues to be ignored.

## Background

As children spend more time in schools than any other venue away from home, they are often seen as key settings in which to address a broad range of public health issues [1]. Indeed, a wide range of interventions have been tried and tested within the school setting with attempts to reveal factors which may have significant impact on the health of children [2,3]. The implementation and sustainability of these interventions is thought to be affected by the organisational context, including the attitudes and beliefs towards health, of staff and managers [4]. This is not inconceivable as this concern has been reported widely in other disciplines. Healthcare professionals’ beliefs have been shown to influence practices regarding obese patients [5] and individual motivations affect implementation of new systems such as shared decision-making [6]. Furthermore, people’s beliefs about the causes of poor health are often consistent with their beliefs regarding the solution [7]. Thus, the health beliefs of school staff may have an important influence over their resultant health promoting behaviour and support for school based health interventions. Indeed, social support from teachers was found to be a significant mediator of positive health behaviour change in a recent school based health intervention [8].

The role of the headteacher in the adoption of interventions is believed to be pivotal. One study revealed that 79% of intervention developers perceived the leadership style and behaviour of the headteacher to be central in the effective implementation of interventions; stressing the importance for those undertaking school based health programmes to develop headteacher support [9]. With childhood obesity being described as an international public health concern [10] and promotion of good mental health of children deemed a high priority [11], there is increasing pressure on schools to implement effective interventions. However, whilst school based interventions have been suggested as demonstrating high public health potential in certain areas such as increasing physical activity [12], addressing health issues whilst maintaining educational standards makes the implementation of school based interventions more challenging.

A degree of autonomy is given to schools regarding the type of health programmes delivered, based on their perception of needs. Therefore, as central decision makers, headteachers’ have potential to impact on local practice and play an important role in the uptake and implementation of school based health interventions. Although there has been some research exploring school staff perspectives on child health [13-16], the majority has primarily focused on classroom teachers and originates from America where the schooling structures are very different. Given the important influence headteachers and school staff can have on the local application of interventions, there is a need for research to better understand their beliefs regarding the health of school children. Gaining understanding in this area can aid practitioners in developing implementation strategies for the effective delivery of evidence based interventions into school settings [14].

This study aimed to explore headteachers’ views on child health and explore how these views may interfere with addressing health concerns of pupils within their school. Gaining further understanding of these insights can assist public health practitioners and researchers when developing and engaging schools in future health interventions.

## Methods

### Participants

Purposive sampling was used to recruit headteachers from primary schools in one local authority. Headteachers from all 84 primary schools in the authority were contacted by email and provided with an information sheet explaining the aims and purpose of the study. This was followed up with a telephone call. Following expression of interest, an interview was arranged at a date and time convenient to the headteacher. If the school headteacher did not wish to participate, this was noted and there was no further communication with the school regarding the study. In a few instances, headteachers requested their deputy or healthy schools co-ordinator take part on their behalf.

### Data collection

This explorative qualitative study used semi-structured interviews. The use of open ended questions facilitated exploration into participants’ views on child health [17]. A semi-structured topic guide, initially validated through two pilot interviews was utilised and interviews explored a variety of topics including aspects of child health considered important to schools and views regarding school based health interventions. Those which related to participants’ views regarding child health are presented in this paper. Interviews were conducted between January and March 2013 and took place in a room within the primary school setting with two researchers present (DC and CB or HD). One researcher (DC) facilitated the interview process and another researcher (CB or HD) provided technical support (digitally recording), observed interaction and made field notes. The second researcher also verbally summarised the key points discussed back to the interviewee at the end of the interview. Participants were encouraged to identify any points of disagreement or expand further on this summary. This provided a form of respondent validation, ensuring an accurate perception of participants views was being obtained [18]. Interviews lasted between 30 and 69 minutes. Using tenets of grounded theory, each interview was followed with a discussion between the two researchers which resulted in adaptations to the interview guide; allowing researchers to explore new concepts as they emerged [19]. Using this approach allowed each interview to influence subsequent data capture and build on understanding as the study progressed [20].

### Data analysis

Each interview involved interaction and responses between the participant and the researcher and was digitally recorded and transcribed verbatim in Microsoft Word. Each transcript was read several times by DC and CB or DC and HD and notes were made regarding general categories and themes to emerge. This allowed researchers to begin immersion in the data [17]. The primary researcher (DC) used open coding, whereby a word or phrase was assigned to each quote, conversation or paragraph, in an attempt to encapsulate the participants’ meaning. A second researcher (CB or HD) independently coded the transcripts in the same manner and then checked the codes for accuracy and consistency with the primary researcher (DC). Agreement between coders was high but any discrepant codes were discussed and agreed upon with a third researcher (HD or CB, alternate to second researcher). This ensured expert validation, enhancing the rigour of the research [18]. Related and reoccurring codes were then grouped together to form main themes, for example codes such as ‘physical and mental health links’ and ‘importance of health on learning’ were brought together under the theme detailing the ‘beliefs regarding the links between child health and learning’. Specific quotations depicting the selected codes were then collated and presented to illustrate the generated themes. After the initial independent coding regular meetings between all three researchers ensured resultant themes, categories and their related quotes were agreed upon.

### Ethics

The study was granted ethical approval by Swansea University College of Human and Health Sciences Research Ethics Committee (CHHS REC) (October 2012). All participants provided informed written consent prior to participating and all personal data was kept separately from transcript data. To ensure anonymity, all participants were given pseudonyms (Pilot 1 and 2, Participant A-Q) and transcripts were anonymised to remove any identifying data [18]. All paper data was securely stored in locked cupboards and electronic data in password protected files on a secure university server.

## Results

19 out of 84 primary school headteachers or their representatives agreed to take part. 13 declined to participate; 9 declined due to busy workloads and an overload of initiatives, 2 due to impending school inspections and 2 gave no reason. 42 schools did not respond and 9 were busy at the time of calling. One school sent their opinions via email. Of the 19 participants who consented, 16 were headteachers, 1 was a deputy headteacher and 2 were healthy school co-ordinators. Twelve of the participants were female and seven were male. Participants belonged to schools where there was a range of children receiving free school meals (3% to 53%;mean 22%). This average of 22% was identical to the non-participating schools.

The interviews revealed a number of contributing factors that could influence the implementation of school-based interventions, such as Governmental policy focus on educational attainment, funding issues and limitations of the school setting itself, which have been described elsewhere [Christian D et al 21]. However this paper focuses on the role Headteachers’ beliefs play in addressing health concerns in their school. Therefore only the associated views are presented herein. Data analysis of these views revealed three main themes:- 1) beliefs regarding the links between child health and learning 2) perceptions regarding the current health of school children and 3) responsibility for child health.

### Beliefs regarding the links between child health and learning

The main view of participants was that improving child health also improved learning and that the two should go hand in hand. Participants regularly remarked on the importance of overall wellbeing and suggested both physical and mental health to be equally important for a child’s ability to learn. As a result, many participants perceived the overall wellbeing of children as a prerequisite to learning and a core factor to be addressed:-

*“Wellbeing is like the corner stone really of learning, isn’t it, I suppose…it’s like Maslow’s hierarchy of needs, you’ve got to have that very basic step in place that the children are… you know, well, the very basics that they’re not hungry…are they fit and healthy, are they able to concentrate”. (Participant O - Headteacher)*

Furthermore, many participants noted a positive link between exercise behaviour and learning in practice, observing children’s engagement and learning was often improved following participation in physical activity. One participant suggested that utilising methods such as short bursts of exercise had been an effective method in helping children focus in the classroom:-

*“Something I’ve seen before which I thought was really useful…was the whole school involved in ten minutes of activity at the same time each day…it meant that sort of midway through the morning the children were being fired up again, we know that it sort of re-sparks the brain and what have you”. (Participant H - Headteacher)*

Despite a common belief among participants regarding the importance of good health for learning and the value placed on promoting physical activity, translating these beliefs into positive health practices, such as engagement with school health interventions presented with many challenges. One such challenge recurrently discussed by headteachers related to prioritising health activities when their primary role is achieving government targets for literacy and numeracy. Suggestions such as the use of cross-curricular approaches to combine health activities and curricular outcomes were put forward as methods of overcoming this issue:-

“*our Year 5 teacher..he uses movement for grammar so for example..they’ll be standing up, he’ll read the sentence and when it gets to where they need to put a full stop in they will do the movement (makes gesture) and then they keep reading and if there are speech marks they will physically do this movement (makes gesture)..so it doesn’t have to be discreet..you can use literacy in your fitness”. (Participant C – School Health Co-ordinator)*

### Perceptions regarding the current health of school children

#### Physical health

Although many participants appreciated the importance of good health for learning, some commented that there were particular health concerns which were not a problem in their school and this had potential to impact on which interventions were adopted as a result. Some remarked on low numbers of obese children in their schools and felt that there seemed to be a mismatch between the high levels of obesity reported among children in the United Kingdom and the low number of overweight or obese children in their school. Consequently, there was satisfaction among some participants that enough was being done in terms of physical activity and nutrition in their school resulting in minimal concerns over obesity:-

*“I was there for 16 years as head, very small numbers of children overweight, which surprised me when I actually thought about it and looked at it, and significant numbers of children doing at least one activity a week. So in terms of that, I think maybe what I referred to ambivalence a bit earlier, it might be a sort of we don’t need to, we’re reasonably satisfied with those health initiatives primarily around healthy eating and physical activity”. (Pilot 2 – Retired Headteacher)*

*“I don’t want to sound complacent because I’m always looking for what is new out there. I’m happy with what we’re doing now and staff would probably tell you it’s enough to get the two lessons of PE, it’s enough to keep up with the afterschool clubs that they run, because it’s quite an extensive afterschool programme that we have, so they will probably say “no, don’t give me anything new”. (Participant F - Headteacher)*

On the other hand, some respondents felt obesity was a key problem needing addressing within their school. Overall, wide variability was demonstrated regarding whether and at what ages weight was believed to be a problem among their school children and this did not seem to be reflective of deprivation. For instance, whilst interventions related to obesity are commonly targeted at more deprived communities, some participants in schools in these areas remarked on a low prevalence of obesity among pupils, whilst others from less deprived schools commented to observe a high prevalence of overweight children in their school:-

*“we’ve still got lots of children here who are unfit and unhealthy you know, I think, I’m sure the school nurse will tell you when it comes to the centres of younger children coming into school with regard to their weight, we see lots of children who’re very, very large children who obviously aren’t living very healthy lifestyles at home”. (Participant B - Headteacher)*

#### Mental health

In keeping with the idea that schools were more likely to adopt interventions in areas perceived as a concern, a number of participants commented that mental health was more of an issue than physical health among children within their school. As a result, some teachers, particularly in deprived communities commented that their initiative focus and priority was predominantly on the mental health of children:-

“*Healthy eating…that is a road we’ve all gone on a long time ago and I suppose as I said to you we take that all for granted now. It’s now that we’re beginning to look at the mental health issues”. (Participant G – Healthy School Co-ordinator)*

Low self esteem and self belief were suggested by some participants as a concern among children in deprived areas; thus improving self esteem of pupils seemed important to these participants. However, whilst mental health was recognised as an important issue, one participant expressed that they were not overly confident in their referral pathways for children with mental health problems such as depression:-

*“I think mental health generally is a huge thing. In terms of self-esteem and building children’s confidence, I think we do a very good job. What happens for those children who may be depressed, do we recognise that and are we able to do anything to help them? I think that our referral mechanisms for children who might have mental health issues is probably something, probably across the country, that is not brilliant…”.(Participant P - Headteacher)*

Hence, this particular school may be more likely to be consider interventions addressing mental health recognition and referral pathways. Whilst individual suggestions were made, on the whole, interventions which took a holistic approach to the health of a child and considered overall wellbeing seemed to be more appealing to schools, than those that took a single component approach:-

“*mental health and physical health, I don’t think you can separate the two, I think it has to be, you have to target the overall child because some children can be physically fit can’t they and very unhappy, yeah so a holistic approach to health as well really”. (Participant C – School Health Co-ordinator)*

#### Gender differences in children’s health behaviour

There was also a difference in participant beliefs regarding the impact of gender on health behaviours. Whilst some did not identify a problem, many commented to be concerned that girls were generally less active than boys, particularly on the playground:-

“*there’s definitely a huge gender gap which is widening…you’ll have the boys playing football and having a scrap because they can’t get on because they think it’s the FA Cup and then you’ll have the girls who are sitting there with their reading books, etc., or sat down on the floor getting piles doing their colouring”. (Participant E - Headteacher)*

*“the boys will just go and play football and they will sort themselves out whereas the girls are more likely to be standing around having a chat and not participating”. (Participant Q – Deputy Headteacher)*

Consequently, it could be seen that interventions which addressed a problem identified by the school as important (in this case gender differences in physical activity), were more likely to be implemented. Indeed, participants who highlighted gender inequalities in physical activity as a concern, advocated a need for interventions to engage girls:-

*“But I come back, what’s the ideal? I think a breadth of things that would appeal to girls.... younger and younger and younger girls now want to talk about fashion and make-up and stuff and maybe not want to run around like mad things. So we’ve got to find some things that appeal to those”. (Pilot2 – Retired Headteacher)*

Indeed, many participants commented on the need to instil physical activity at a young age, particularly among girls in order to encourage positive health behaviours*.*

#### Socioeconomic differences in children’s health

Many participants believed the health issues faced by children differed between schools in deprived and affluent areas and participants in deprived schools commented to adopt different initiatives as a result:-

*“this is a community school and..obviously an area of poverty and we just have to weigh up the pros and cons and I suppose again with any initiative that you’d want to put together, you would look at the community in which you were offering it to if you see what I mean? Slightly different if you offer some west community to perhaps a community here, you know, and I think those are the sorts of things you need to look at”. (Participant G - Headteacher)*

Participants commented on the importance of understanding the community around the school when planning health interventions, suggesting communities are in need of different approaches. As a result, the type of support from external health intervention providers may need to differ according to particular issues faced by children in these schools. For example, financial support (such as provision of subsidised activities and transport) may be required in deprived areas but support for time restricted parents may be needed in more affluent areas:-

“*I’ve worked in a number of different areas and what I’ve recognised is that interventions, although there seems to be more financial input into areas which are designated socially disadvantaged, or economically disadvantaged, and I challenge that really because I think that there is a disadvantage in being in a so-called ‘affluent’ area, because obviously children who come from professional working families there perhaps is less time if you like there, it’s not a financial aspect, difficulty, but there are different pressures and different challenges, so I question that if you like, that you know, I do think there’s an inequality there”. (Participant H - Headteacher)*

These responses suggest that participants feel there should be a greater understanding of local factors, rather than consideration of the socio-economic location of the school alone when delivering health interventions.

### Views regarding responsibility for child health

#### School versus parental influence

One strong theme developing throughout the interviews was that of responsibility and whether a child’s health should be addressed predominantly by parents or within schools. Many participants believed as a school they had a level of responsibility and duty with regards to children’s health. However, the level of responsibility participants attributed to themselves and the school varied. Some attributed a lot of responsibility to parents, whilst others revealed that they took on a greater health role due to their belief that children may not otherwise get the opportunity for a healthy lifestyle. Overall, there was a widespread concern that this responsibility was becoming too much at the sacrifice of the teaching and learning role of the school. Furthermore, a feeling of uncertainty regarding what level of responsibility the school should have over the health of children was evident throughout responses. Some argued that social aspects of health were becoming more prevalent in schools, particularly in deprived areas, leaving staff to feel in a moral dilemma as to whether it was their responsibility to address this. One participant expressed that teachers did not want to raise this issue for fear of being considered uncaring or negligent and there was a clear concern where the line would be drawn. Participants discussed the increased health roles taken on by schools at present, particularly in more deprived areas, advocating the urgent need for better partnerships between school and parents when addressing health:-

*“My wife worked the other side of town and, as she said, ‘We give them breakfast, we teach them how to brush their teeth, we teach them what fruit is and how to eat it, we teach them how to hold a knife and fork. We’ll be the one there before long in getting them out of the bed and dressing them’. That’s not to say that the things that we do in school are not important, but surely there’s a partnership?” (Participant P - Headteacher)*

The responsibility attributed to parents for children’s health also differed among participants. Some participants mentioned the positive role parents play within the school in terms of being supportive and keen for their children to be given opportunity. However, many believed parental engagement regarding school health initiatives was often minimal. One participant suggested this may be due to parents believing schools to be providing sufficient opportunities for healthy behaviours*,* whilst others suggested a variety of parental factors could impact on the situation, including the parents own experience of school:-

*“I don’t know, I mean, you know engaging parents is difficult, it’s really difficult and we’ve been working really hard at that, engaging of parents, and you know making slow progress with it. A lot of parents in this area, you know 50% of our children are in a town in (name of deprived area) and they’ve had bad experience at school themselves, so you know they don’t want to even come in to school”. (Participant A - Headteacher)*

Teachers believed this lack of parental engagement often resulted in a lack of opportunity for some children in adopting positive health behaviours such as accessing physical activities. As a result, many believed that what was being done within school to address health was contradicted by parents when children were at home. Lack of time, parental convenience and lack of parental knowledge were also offered as reasons for this lack of engagement. However, lack of parental knowledge was debated as one participant believed that parents had the knowledge and were aware of what constitutes a healthy lifestyle. Nonetheless, perceived that parents found it easier to engage their children in sedentary or unhealthy behaviours:-

*“don’t tell me anybody doesn’t know that having a dummy soaked in Ribena all day long with sugar doesn’t rot your teeth. People might not come out of school with GCSEs and A levels but I can assure you they don’t come to school dull, they know exactly what’s what when it suits, but it keeps the kids quiet at times”. (Participant N - Headteacher)*

Beliefs regarding parental influences and responsibility seemed to impact on how much of a health role the school felt they should take on as a result. These beliefs could affect the way in which and whether or not schools choose to implement an intervention, with some headteachers feeling disinclined to take on certain health roles:-

*“I think intervention programmes have been encouraged at home, that hasn’t worked, so let’s tie it into school and make it school’s responsibility and like I say, it’s that that I resist really is that because, like I say, we’re about learning and teaching, and yes of course learning to teach clean your teeth is an important skill but it’s not one that we need to be doing everyday if you like, and yeah, I think it’s the responsibility of the home really”. (Participant H - Headteacher)*

#### Role of society

In addition to parental influences, many participants commented on the part played by society, suggesting societal changes such as concerns over neighbourhood safety, changes to parental working hours and increased use of technology were influencing children’s health:-

*“..are people comfortable leaving their kids out in the streets, down in the woods you know, where we used to play, and out from dusk till dawn you know, I don’t think parents are quite happy in doing that anymore, and when lots of children now go home the parents are actually going to work and therefore they become involved in their own little world, Xbox, PlayStation, and they can play their friends online so they are socialising without actually getting out and wandering the streets”. (Participant N - Headteacher)*

Thus, the schools felt too much responsibility was being placed at their door, when in fact parents and society in general needed to take much more responsibility. Many felt improving children’s health should be a partnership between schools, government, parents and society and this was not being effectively addressed.

## Discussion

This study provides insight into headteachers’ and staffs’ perspectives regarding the health of primary school-aged children. Findings show that school staff do believe that children need to be healthy for learning and many want to promote health in their school. Interventions which take a holistic approach to health and wellbeing and consider learning outcomes are more likely to be appealing to schools and engage school staff. However, interventions which target problems which they feel are not present in their school are less likely to be adopted, implemented or sustained. Instead, programmes which target problems perceived as relevant to their school are likely to have greater support. Thus, interventions need to be adaptable to address local barriers and a “one size fit all” approach will not be suitable for school based health interventions. Finally, schools do not regard child health as solely their responsibility but believe interventions need to look wider at engaging parents and society to take a shared responsibility for children’s health, in partnership with schools.

### Links between child health and learning

Supporting the need for better links between health and education, previous work has found positive associations between a variety of health related behaviours and academic outcomes. As such, researchers have proposed that health and education are highly interrelated and improving health and academic outcomes should be a joint goal of health and education specialists [22]. However, research regarding the link between different health behaviours such as physical activity and academic outcomes remains far from conclusive [23] and more research is needed to evaluate the impact of health interventions on educational outcomes. Nevertheless, given the common shared belief among many participants in this study that good health is important for learning, it can be expected that if schools can see educational benefits from health programmes, they will be more likely to be implemented and sustained. Furthermore, better implementation of interventions may occur if health promotion practitioners can demonstrate how aspects of the curriculum can be covered within school-based health interventions.

### Current health of school children

Perceptions regarding the predominant health issues faced by children within their schools varied and participants were more likely to be supportive of interventions addressing those issues perceived as a concern. If the transtheoretical model of behavioural change is applied to this situation, it can be seen that in order to contemplate behaviour change, in this case, adoption of a particular health intervention, headteachers and staff must first perceive that there is a health problem among children in their school [24]. Therefore, if headteachers and healthy school co-ordinators do not perceive particular issues such as obesity as a problem in their school, they will be less likely to consider interventions addressing this concern. This has important implications as research has shown that a mismatch can occur between perceptions of children’s health, such as children’s weight status, and their actual weight status [25]. Therefore, whether staff perceptions of the current health status of children are reflective of the actual health status of children within their school would need to be confirmed through objective measurement, highlighting the importance of local needs assessment. Better evidence is needed regarding the health and wellbeing of children to enable comparison locally, regionally and nationally and assist local stakeholders concerned in developing better partnerships and planning interventions based on local need.

An interesting point raised among participants within several schools was the shift of initiative focus from physical health towards mental health of pupils. The importance of good mental health was mentioned by numerous participants and the majority of participants placed value on a holistic approach to child health. On a practical level, recognition and referral of mental health problems was highlighted as a concern by some teachers. This has also been identified among primary school teachers in previous research where a lack of support and guidance was highlighted in enabling teachers to deal with mental health concerns among pupils [14]. Researchers have advocated training for teachers regarding identification and timely referral of mental health problems [14,26]. However, if teachers are to be encouraged to take a wider role in mental health, releasing staff for training may be an issue in curricular time and potential ways of overcoming this need to be thought through. A deeper insight into the mental health and wellbeing needs of primary school pupils is advocated. Furthermore, incorporating wellbeing measures into evaluation of school based health interventions would help ensure a more rounded understanding is gained.

Gender differences in health behaviour, particularly physical activity, were remarked upon by many participants in this study and lower levels of activity in girls have also been reported when quantitative examination of school children has been undertaken [27,28]. The perceived emergence of this inequality by a number of participants, particularly at younger ages, suggests that many headteachers and healthy school co-ordinators may be more likely to consider physical activity interventions which offer increased opportunities for girls. Indeed, whilst using methods such as the introduction of non-traditional sports such as street-dance have been suggested as appealing to adolescent girls [29], they are yet to be employed to the same degree in primary school children, and may provide a useful avenue for increasing engagement. Future interventions should aim to evaluate differences in the impact of interventions on boys and girls both qualitatively and quantitatively to provide greater insight into barriers and change mechanisms. Without such consideration in health promotion interventions, gender inequalities may widen. Furthermore, if objective measurement confirms these health perceptions, greater arguments for allocation of resources can be provided and issues pertinent to the school, different age groups and gender can be better addressed. This would avoid the blanket application of interventions, without local consideration of need.

Consideration of socio-economic differences in health behaviour and health needs was also suggested as important and gaining understanding of the community around the school was advocated. This understanding will ensure the correct approach is utilised to address particular health problems. Indeed many believed allocation of resources based solely on the location of the school was not fair or the most effective approach. Thus, gaining a holistic understanding of the interplay between the many influences over a child’s health in that particular area will be important in helping to justify provision of resources and type of approach undertaken. Indeed, while some health issues are present in large numbers of the population, different approaches and resources may be needed by communities when responding to these issues and developing health programmes without local understanding of needs is likely to result in failure [30].

### Responsibility for child health

This study has revealed a clear concern regarding responsibility for health. Whilst many issues discussed by participants are reflected in the socioecological model for health [31], there was a wide variability in beliefs regarding how much responsibility should be attributed to each level. This has important implications as inherent to many theories of behaviour change is the notion of self-efficacy [32]. The Stages of Change, Social Cognitive Theory and Health Belief Model attach importance to the notion that people must believe in their ability to change a particular problem to both initiate and sustain behaviour change [33]. Thus, if responsibility for particular child health issues is believed to be largely outside the school’s control, headteachers and staff may be disinclined to take on interventions. This issue of responsibility has been highlighted in a recent review which demonstrated variation in parental opinion regarding who is responsible for health issues such as child weight management, with a large number of parents believing schools to be largely responsible [34]. Whilst teachers in this present study acknowledged their important role in health promotion, many believed there was too much responsibility being placed on schools. Some participants felt an increasing need to provide opportunities for positive health behaviour due to concern that it may be lacking in the home environment; again concurring with views of teachers in a previous study [15]. Several perceived a lack of parental engagement and support necessary for encouraging positive health behaviours among children. This is concerning; for whilst research is inconclusive regarding the effect of parental involvement in interventions[35], parental support and family environment are believed to play a key role in influencing children’s health behaviours [36,37]. Thus, establishing reasons for this lack of engagement, localising which groups are more of a concern, and finding effective methods of increasing this engagement are needed. For instance, some qualitative research has suggested parents in deprived communities may be more reluctant to encourage certain activities due to a concern they could not logistically or financially provide for long-term continuation of these activities [38]. Developing a shared understanding of these barriers is important as children of primary school age generally have limited control over their physical activity options and food choices [39]. It is important that both teachers, parents and practitioners are aware of their shared responsibility to health and that the onus isn’t left on one party alone. Improving relationships and increasing collaboration of all involved will facilitate shared responsibility to addressing child health issues.

Social influences on health were also believed to play a part, with participants suggesting neighbourhood safety and increased use of technology in today’s society resulted in more sedentary children. Neighbourhood social factors such as safety has been shown to significantly influence physical activity levels of children [40]. Therefore, incorporating parental and societal aspects into interventions may be seen as important to many staff when addressing particular health behaviours. Furthermore, dependent on their beliefs, some headteachers and staff may be more attracted to these interventions as they adopt a shared approach to addressing health, rather than attributing all responsibility to the school. It is likely that initiatives attributing responsibility to one sector or individual alone will not be productive [38].

Staff perceptions can provide useful insights into the health of school children and the critical role played by the headteacher cannot be overlooked. However, it is also important to recognise that there is potential for those children who attend school where a headteacher may be less interested in health, to be at a disadvantage. Consequently, more evidence is needed regarding the health of children and future research should consider the views and experiences of other responsible adults, alongside the headteachers’ viewpoint. If neither headteachers, staff or parents perceive a particular health issue as their responsibility or do not have the resources or skills to address those issues, there is potential that certain health issues become neglected. Parents, teachers and all those with the potential to influence child health need to work together to understand the challenges and facilitators inherent in promoting the health of primary school children. Moving evidence-based interventions into real world settings is a complex task and the sustainability of interventions may be highly dependent on both the school and parental support of the intervention. Therefore, it is important that partnerships strengthen, to optimise the health and wellbeing of school aged children.

### Strengths and limitations

The findings presented need to be taken in the context of the study’s strengths and limitations. The study has strength in adding to an important but under-researched area in the field of child health. The use of qualitative interviews allowed researchers to gain a deeper insight into participants’ views regarding health in school children and the methods of respondent and expert validation increased the ‘trustworthiness’ of these findings. However, as participants volunteered to take part, there may have been a selection bias introduced with recruited participants having a greater interest in children’s health and wellbeing. In addition, limited data was gathered regarding participants’ age, culture and years of teaching, which have the potential to inform people’s personal beliefs. Furthermore, whilst staff beliefs may be important in influencing their health promoting behaviour, perceptions alone do not always mirror behaviour, and it is important to take other factors into account [33]. Whilst staff may believe in the need to promote health, wider influences such as government leadership, curricular pressure and funding may also affect intervention implementation [13,15,16]. It would be useful for further research to explore these influences and gain the perceptions of wider stakeholder groups with responsibility for child health, including parents.

## Conclusion

- Understanding the health beliefs of school staff is crucial when designing interventions as school staff need to perceive a particular health issue to be a concern for their pupils before considering implementing a corresponding intervention. Greater information is needed at the school level in order to highlight and direct resources at areas of need.
- School staff identified clear impacts of health and wellbeing on educational outcomes and would be more likely to engage with and sustain interventions that addressed these components with more of a holistic approach.
- Furthermore the issue of responsibility is an interesting avenue to explore. As if neither staff, parents or pupils perceive particular health issues as their responsibility or do not have the resources, skills or support to address those issues, there is potential that certain child health issues will become neglected.
